# Structural Insights into Notum Covalent Inhibition

**DOI:** 10.1021/acs.jmedchem.1c00701

**Published:** 2021-07-22

**Authors:** Yuguang Zhao, Fredrik Svensson, David Steadman, Sarah Frew, Amy Monaghan, Magda Bictash, Tiago Moreira, Rod Chalk, Weixian Lu, Paul V. Fish, E. Yvonne Jones

**Affiliations:** † Division of Structural Biology, Wellcome Centre for Human Genetics, University of Oxford, Oxford OX3 7BN, U.K.; ‡ Alzheimer’s Research UK UCL Drug Discovery Institute, University College London, Cruciform Building, Gower Street, London WC1E 6BT, U.K.; § Centre for Medicines Discovery, University of Oxford, Oxford OX3 7DQ, U.K.

## Abstract

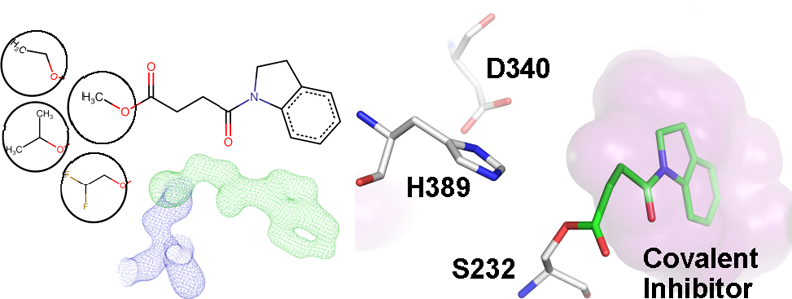

The carboxylesterase Notum hydrolyzes a palmitoleate moiety from Wingless/Integrated(Wnt) ligands and deactivates Wnt signaling. Notum inhibitors can restore Wnt signaling which may be of therapeutic benefit for pathologies such as osteoporosis and Alzheimer’s disease. We report the identification of a novel class of covalent Notum inhibitors, 4-(indolin-1-yl)-4-oxobutanoate esters. High-resolution crystal structures of the Notum inhibitor complexes reveal a common covalent adduct formed between the nucleophile serine-232 and hydrolyzed butyric esters. The covalent interaction in solution was confirmed by mass spectrometry analysis. Inhibitory potencies vary depending on the warheads used. Mechanistically, the resulting acyl-enzyme intermediate carbonyl atom is positioned at an unfavorable angle for the approach of the active site water, which, combined with strong hydrophobic interactions with the enzyme pocket residues, hinders the intermediate from being further processed and results in covalent inhibition. These insights into Notum catalytic inhibition may guide development of more potent Notum inhibitors.

## Introduction

Secreted Wingless/Integrated(Wnt) morphogens are key components of Wnt signaling.^1^ Wnt proteins are post-translationally modified with a palmitoleic acid (PAM) moiety attached to a conserved serine (e.g., human Wnt3a S209). This lipid plays a vital role in Wnt’s binding to their primary receptors, which are members of the Frizzled family.^2,3^ The lipid modification is carried out by a membrane-bound *O*-acyl transferase family member called porcupine.^4^ The modification can be reversed by an extracellular carboxylesterase, Notum, which hydrolyzes the lipid from Wnt ligands^5,6^ to maintain appropriate levels of Wnt signaling.

The evolutionarily conserved Notum enzyme plays many important functions by modulating Wnt signaling. In *Drosophila*, Notum coordinates synapse development.^7^ In planarians, flat worms have the ability to regenerate themselves from minuscule body parts, Notum is important for head regeneration,^8^ and it is the only gene differentially expressed at the wound site.^9^ In zebra fish, Notum regulates motor axon guidance.^10^ In mammals, Notum regulates fat metabolism,^11^ liver glucose homeostasis,^12^ colon stem cell aging,^13^ bone strength,^14,15^ dentin morphogenesis,^16^ tracheal development,^17^ catagen progression in dermal papilla,^18^ and ghrelin hormone deactivation.^19^ Notum also plays a key role in adult brain ventricular–subventricular zone neurogenesis.^20^ Notum inhibitors can rejuvenate aged colon stem cells,^13^ increase cortical bone thickness and strength,^15^ and increase adult neuronal progenitors^20^ and are being investigated for potential treatment of neurodegenerative pathologies such as Alzheimer’s disease,^21−28^ in which Wnt signaling is commonly downregulated.^29,30^ More recently, Notum has been identified as a key mediator for adenomatous polyposis coli (Apc)-mutated tumor cell fixation and tumor formation, while Notum inhibitors abrogate the ability of Apc-mutant cells to expand.^31^ These pieces of evidence highlight the importance of Notum as a novel target for drug discovery.

One major challenge in drug discovery is achieving high potency and selectivity. Chemical compounds forming a covalent bond with an enzyme nucleophilic residue may have enhanced selectivity and potency with fewer off-target effects and have the potential to be used in smaller doses and with less frequent dosing than noncovalent inhibitors. Despite initial concerns about their safety, development of covalent inhibitors has re-emerged as an effective approach for novel drug discovery.^32,33^ Approximately, one-third of enzyme targets have FDA-approved covalent drugs.^34^ For example, aspirin, the most widely used medication in the world, covalently modifies cyclooxygenase by acetylating the serine-530 residue near the active site.^35^

Recently, a class of irreversible Notum inhibitors has been discovered by activity-based protein profiling,^36^ although the exact biochemical mechanisms remain to be defined. Starting from the available structural information on Notum, we used both virtual and crystallographic screening to identify novel covalent inhibitors. Here, we report methyl 4-indolinyl-4-oxobutanoate and its derivatives as covalent Notum inhibitors and characterize the inhibition mechanism at an atomic level.

## Results and Discussion

### Virtual and Crystallographic Screening Identified a Covalent Inhibitor

**Figure 1:**
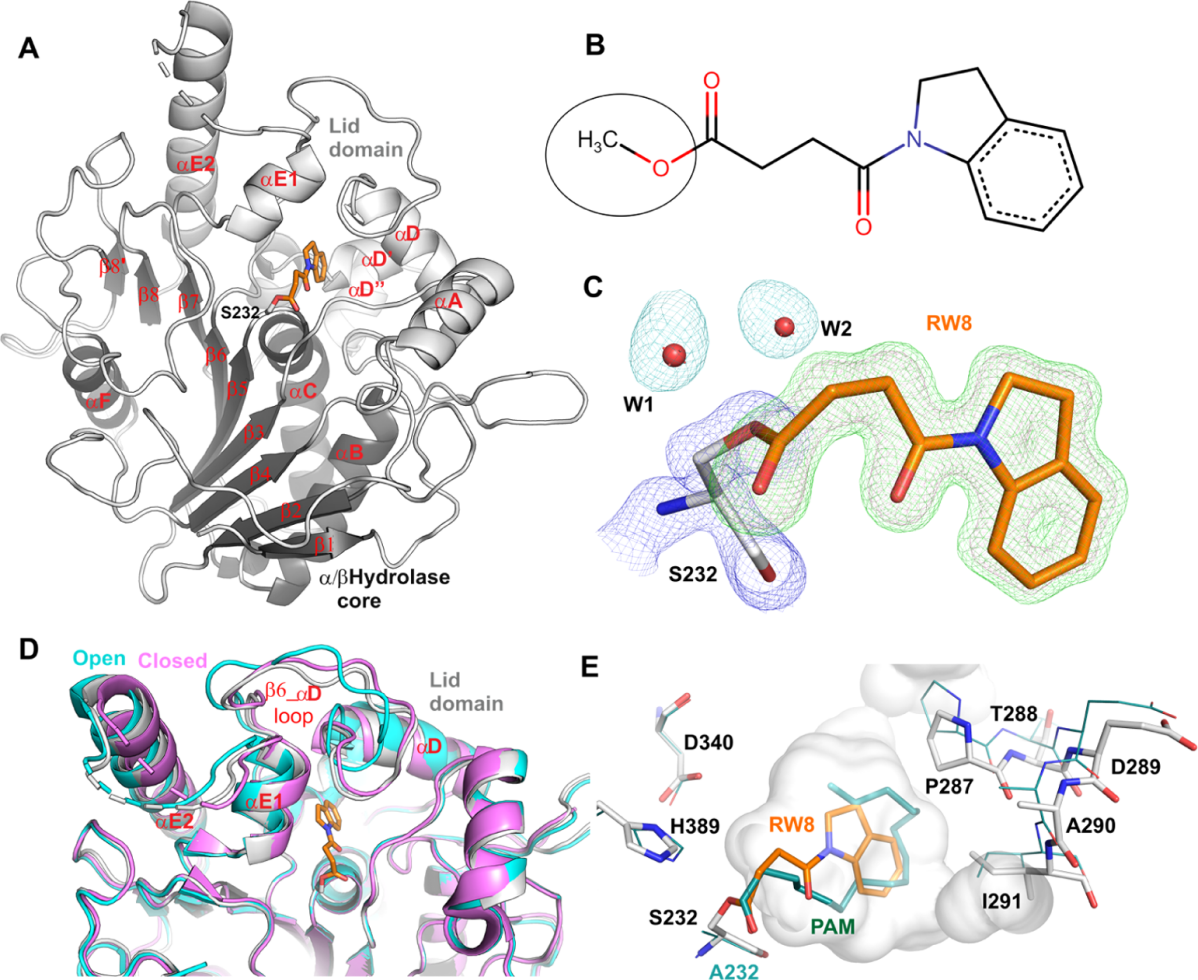
(A) Cartoon representation of Notum complexed with methyl 4-indolinyl-4-oxobutanoate (**1**) (PDB code 7ARG). The α/β hydrolase core is colored in dark gray, while the moveable lid domain is colored light gray. (B) Chemical structure of **1**. The circled methyl ester group is hydrolyzed by Notum. (C) Electron density of the Notum nucleophile residue S323 |2*F*_O_ – *F*_C_| map (in blue mesh contoured at 1.5σ) bonds with the **1** methyl ester-hydrolyzed form (RW8) |*F*_O_ – *F*_C_| omit map contoured at 3σ (green mesh) and 5σ (pink) and two active site waters |*F*_O_ – *F*_C_| omit map contoured at 3σ (teal mesh). (D) Superimposition of the Notum moveable lid domain of **1** complex with the Apo structures in open (PDB code 4UYU, in cyan) and the closed (PDB code 4UZ1, in purple) conformations. (E) Overlay of **1** complex with the natural substrate lipid *O*-palmitoleoyl (teal) (PDB code 4UZQ) complex. The surface of the Notum-**1** pocket is outlined in gray. The catalytic triad and the pocket bottom forming residues exhibiting conformational changes are shown as sticks.

Notum is a druggable target for Wnt signaling modulation.^23^ We have previously determined a number of structures of Notum inhibitor complexes.^21,22,24,25,27,28^ All of these inhibitors bind noncovalently at the Notum enzyme catalytic pocket, where the natural substrate PAM binds. For searching covalent inhibitors, we used this noncovalent inhibitor binding information to limit compound docking into the enzyme active site. A virtual library of approximately 1.5 million compounds (ChemDiv) was filtered to generate 534,804 candidates for docking with a Notum structure (PDB code 6T2K) by Schrödinger Glide SP^37,38^ (Experimental Section). Resulting 1330 compounds with score −9 or better were subject to manual inspection and availability check. Finally, 952 compounds were purchased and experimentally screened using a cell-free trisodium 8-octanoyloxypyrene-1,3,6-trisulfonate (OPTS) biochemical assay.^22^ A total of 31 compounds with Notum IC_50_ < 500 nM were subject to Notum crystal soaking to screen. The soaked crystals were harvested, and diffraction data were collected at the Diamond Light Source. The structures were determined by molecular replacement and refined with Refmac to screen for difference map peaks (Experimental Section). Chemical structures were then fitted into the difference maps with the aim of identifying covalent binders. Methyl 4-indolinyl-4-oxobutanoate (**1**) was identified as the only covalent binder to emerge from the screen (Figure 1). The **1** soaked crystal diffracted to 1.2 Å resolution with a *P*2_1_2_1_2_1_ space group containing one enzyme-inhibitor complex in an asymmetric unit (for data collection and refinement statistics, see Supporting Information, Table S1). The overall structure maintained the characteristic apo Notum fold (Figure 1A), with a α/β hydrolase core domain (β1-8 strands and αB, C, and F helices) and a moveable lid domain (αA, D, and E helices). **1** is composed of an indole ring linked to an oxo-butyric acid methyl ester (Figure 1B). The methyl ester-hydrolyzed **1** (with PDB identifier RW8) is an excellent fit in the strong electron density that extends from the nucleophile residue S232 (Figure 1C). The electron density for the RW8 is of equally high quality to that of the surrounding Notum amino acids. The unbiased omit map shows a strong clear density even at a high, 5σ, contour level, suggesting full or near full inhibitor occupancy (Figure 1C). Superposition of the inhibited structure with an apo Notum structure (PDB code 4UYU) revealed very little variation in the enzyme core domain with a root-mean-square deviation (rmsd) of 0.5 Å (for 220 equivalent Cα atoms). The movable lid domain sits between the “open” (PDB code 4UYU) and “closed” (PDB code 4UZ1) conformations with obvious movements of the flexible β6_αD loop (Figure 1D). When superimposed with the natural substrate, PAM-bound, structure (PDB code 4UZQ), the RW8 overlaps with the PAM within the enzyme pocket (Figure 1E). The indole ring is located toward the bottom of the pocket, while the butyric acid head group is well-aligned with the PAM head (Figure 1E). Residues that form the base of the pocket (P287–I291) are positioned closer to the RW8 indole ring than in the PAM-bound structure. For example, P287 Cα has moved 1.7 Å toward the pocket center (Figure 1E) to optimize interactions with the RW8 indole ring, which extends less far into the pocket than PAM.

### Mass Spectrometry Detection of Notum **1** Covalent Binding

**Figure 2:**
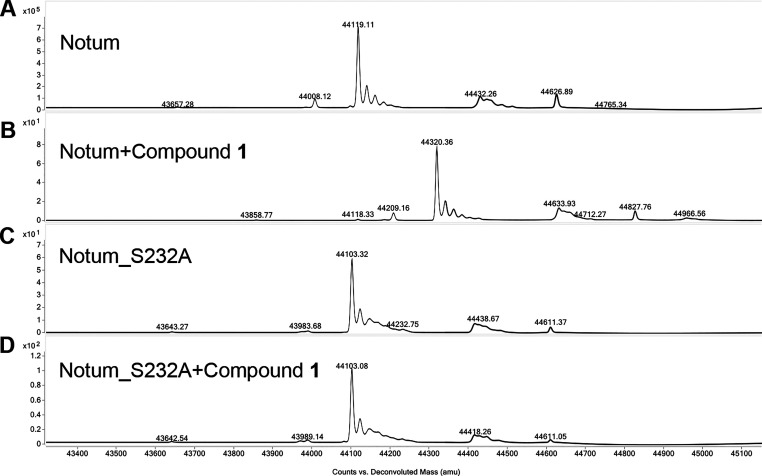
(A) Notum protein mass spectra from ESI-QTOF after chromatographic separation with in-line uHPLC. (B) Notum treated with **1**. (C) Notum with S232A mutation. (D) S232A mutant Notum treated with **1**.

The crystal structure revealed that **1** forms an acyl-enzyme intermediate. However, the crystals were grown and soaked under low pH (pH 4.2) and high salt (1.5 M ammonium sulphate) conditions. To measure covalent bonding and stability under near physiological conditions, we incubated Notum (deglycosylated) protein with **1** in buffer containing 10 mM Hepes, pH 7.4, 150 mM NaCl at room temperature for 1 h. The mixture was then subjected to ultrahigh-performance liquid chromatography (uHPLC) reversed-phase chromatography separation, followed by electrospray ionization quadrupole time-of-flight (ESI-QTOF) mass spectrometry analysis. Notum (core sequence, see Experimental Section) shows a *m*/*z* of 44119.11 Da (Figure 2A), which matches the expected molecular mass (calculated protein MW plus glycan cores). There are some other minor peaks that may reflect incomplete removal of glycans. The mass of the **1** treated Notum shows a major peak of 44320.36 Da (Figure 2B), a 201.25 Da increase compared to untreated Notum. The calculated MW of **1** is 233.27 Da, while its methyl ester-hydrolyzed form (RW8) is 201.22 Da. This suggests that the methyl ester-hydrolyzed **1** is covalently bound to Notum. A minor peak of 44118.33 Da with an intensity <10% relative to the main 44320.36 Da peak suggests that the reaction may be reversible to a small degree. To establish whether the covalent link is dependent on the nucleophile serine residue, we further used the Notum S232A mutant as a control, observing a mass of 44103.32 Da (Figure 2C). The decrease of 15.79 Da compared to the wild type is consistent with the expected serine to alanine mutation. However, when treated with **1**, there is no mass change (Figure 2D). These data show that **1** can bind to Notum covalently in solution close to physiological conditions, and the binding is dependent on the nucleophile S232 residue.

### Thermal Shift Assay Detects Notum **1** Interaction

**Figure 3:**
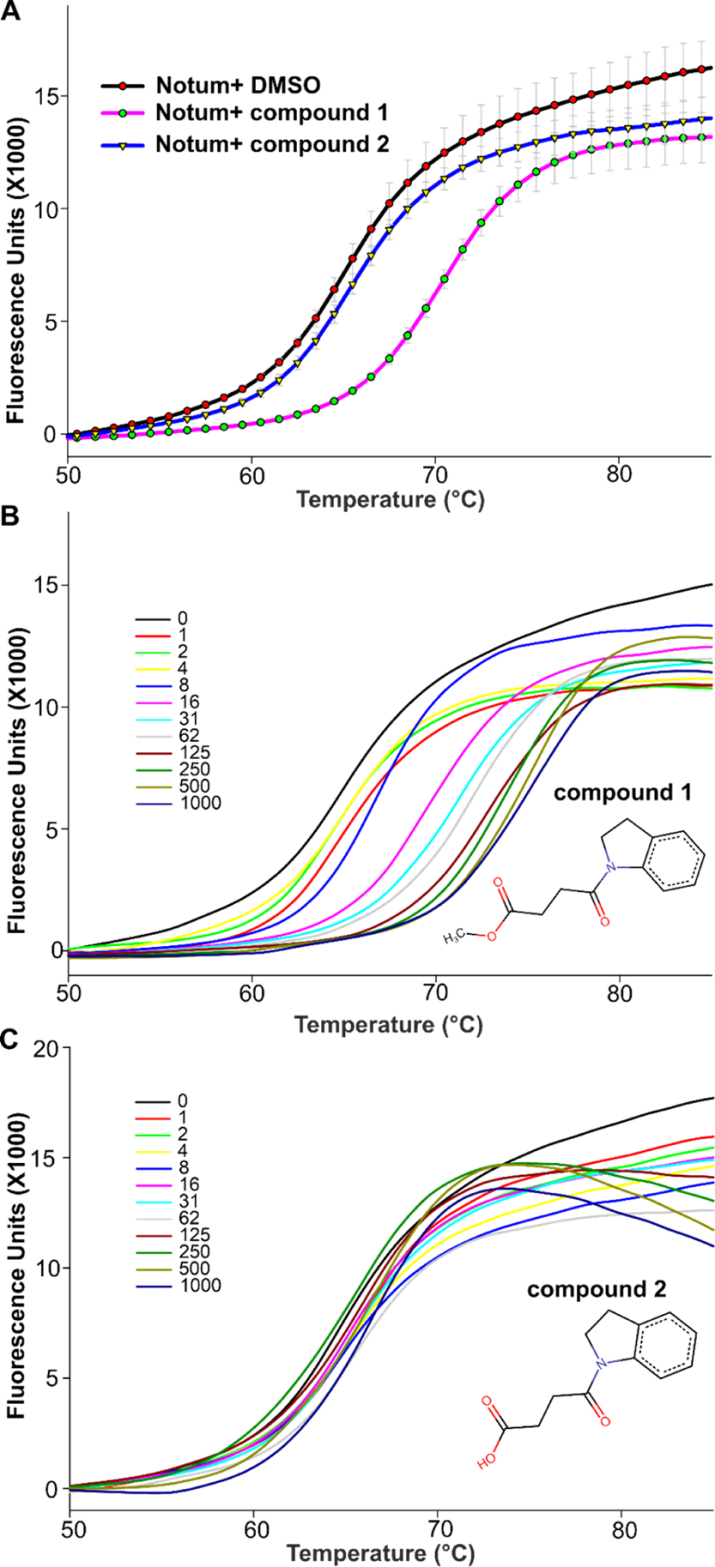
(A) Thermal shift assay showing the melting curve of Notum (glycosylated) with **1** (magenta line) and **2** (blue) at concentrations of 31 μM and control vehicle (black). The error bars represent standard deviation from triplicate measurements. (B) Thermal shift melting curve showing a dose-dependent response (1–1000 μM) for **1** and (C) **2**. The chemical structures of the compounds are shown at the bottom right of the panels.

To further characterize Notum **1** binding, especially regarding the importance of the methyl ester warhead of **1**, we used a thermal shift assay (also known as differential scanning fluorimetry) to compare the binding properties of **1** with its methyl-less acid form, 4-indolinyl-4-oxobutanoic acid (**2**). The thermal shift assay is a reliable label-free strategy to detect small molecule–protein binding by measuring protein melting temperature (*T*_m_) changes.^39−41^ A Δ*T*_m_ of 2 °C or more indicates a small molecule binding to the target protein.^42^ Notum was incubated with each compound in the presence of a fluorescent indicator, orange G, and the melting curves were recorded. **1** and **2**, despite only differing by a single methyl group, yield highly different Notum melting curves (Figure 3). At a middle range concentration of ∼30 μM, **1** yields a Δ*T*_m_ of 5 °C, while **2** yields a Δ*T*_m_ of only 1 °C (Figure 3A), indicating that **1** is a strong Notum binder, while its acid form **2** is only a very weak binder. To establish the dose-dependent thermal shift responses, we performed a twofold serial dilution of the compounds. For **1**, the responses were dose-dependent and start to show significant changes (Δ*T*_m_ 2 °C) from 8 μM, with a max Δ*T*_m_ of 10 °C at 1 mM (Figure 3B), while **2** only shows a max Δ*T*_m_ of 2.5 °C at 1 mM (Figure 3C). The results suggest that the methyl ester warhead is important for Notum binding. Interestingly, the Notum protein used here is a fully glycosylated form, and the compound-free *T*_m_ 65.5 °C observed here is 1 °C more than we previously reported for Endo F1 deglycosylated Notum.^27^ This is consistent with the hypothesis that glycosylation makes proteins more stable.

### Warhead Requirements for Covalent Inhibitors

**Figure 4:**
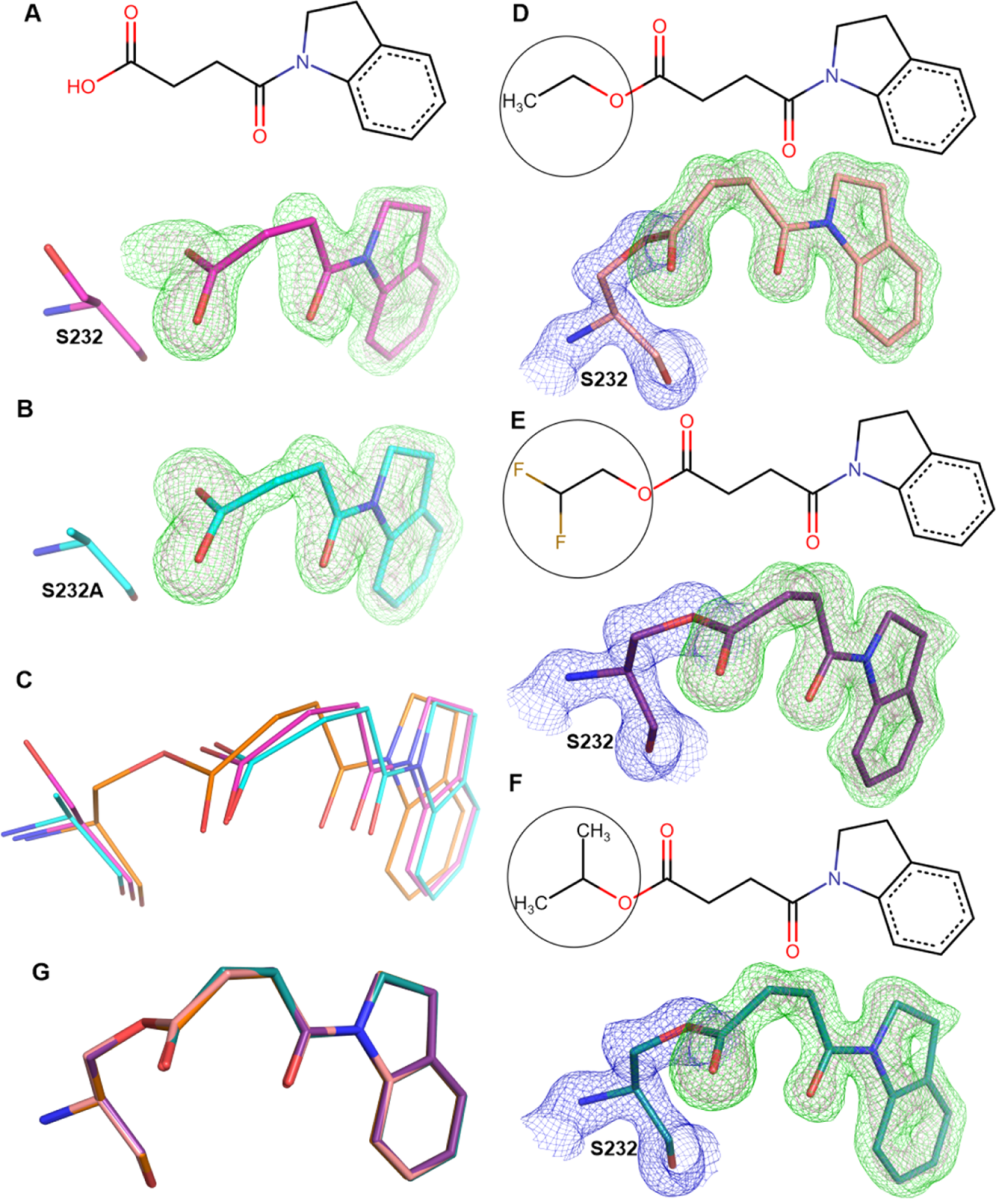
(A) Chemical structure of **2** and its electron density |*F*_O_ – *F*_C_| omit maps contoured at 3σ (green mesh) and 5σ (pink). PDB code 7B37. (B) Notum S232A mutant in complex with **2**. PDB code 7B3F. (C) Alignment of the Notum complex with **1** and **2** and the mutant Notum with **2**. (D) Chemical structure and electron density omit maps for **3** (same contour level as in A), with nucleophile S232 |2*F*_O_ – *F*_C_| map in blue mesh contoured at 1.5σ, PDB code 7B2V and for **4** (E) and **6** (F) with PDB codes 7B2Y and 7B2Z, respectively. (G) Alignment of covalently bonded structures of **1**, **3**, **4**, and **6** based on superpositions of the enzyme-inhibitor complexes.

The thermal shift assay suggested that the methyl ester warhead of **1** is very important, while the acid form **2** is only a very weak binder. To investigate the structural basis for this observation, we first soaked Notum crystals with **2** and determined a 1.4 Å resolution structure (Supporting Information Table S1). The electron density for the acid head and indole ring electron densities were obvious, but there was little or no density for the butyric carbons, indicative of a degree of disorder (Figure 4A). The electron density for the butyric carbons was restored when **2** was soaked into Notum S232A mutant crystals, consistent with the disorder arising from S232 sterically hindering optimal positioning of the acid form (Figure 4B). Superposition of these two complexes with the **1** complex shows the interplay between nucleophile position and covalent or noncovalent inhibitor binding, with a shift in the S232 Cα of 0.6 Å between complexes involving **1** or **2** (Figure 4C). These data further demonstrate that the methyl ester warhead is important for forming a covalent bond with the nucleophile S232.

**Scheme 1:**
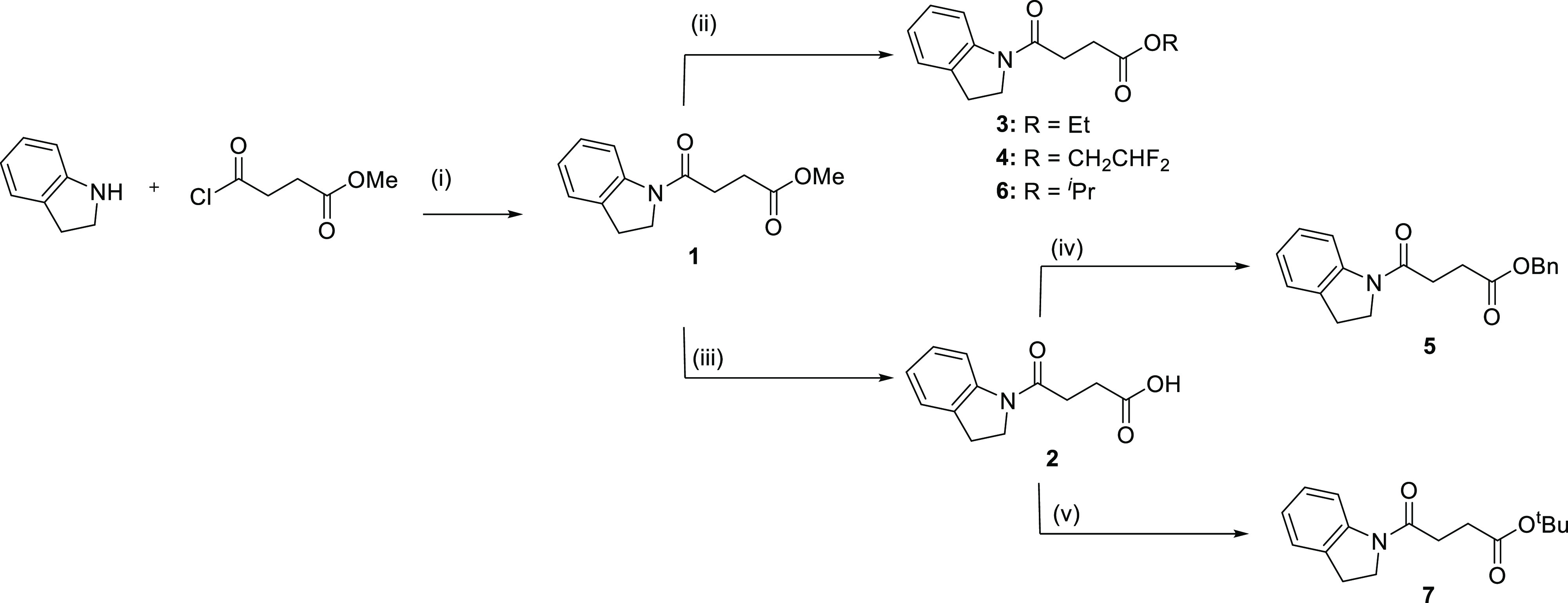
Reagents and Conditions (i) DMAP, pyridine, 50 °C, 3 h; (ii) *p*-TSA, ROH, 40 °C, 18 h; (iii) LiOH_(aq)_, MeOH/THF/H_2_O, (2/1/1), RT, 4 h; (iv) BnOH, WSCDI, DMAP, Et_3_N, DMF, 70 °C, 18 h; (v) conc. H_2_SO_4_, MgSO_4_, ^*t*^BuOH, RT, 18 h.

To further investigate the warhead requirements, we synthesized a set of 4-(indolin-1-yl)-4-oxobutanoate ester derivatives (Table 1). Similar to **1** and **2**, compounds **3–7** were prepared using established synthetic methods from readily available starting materials (Scheme 1). The reaction of methyl 4-chloro-4-oxobutanoate with indoline gave methyl ester **1**. Hydrolysis of the ester of **1** with lithium hydroxide gave the corresponding acid **2**. Acid-catalyzed transesterification of **1** with ethanol, 2,2-difluroethanol, or *iso*-propanol gave **3**, **4**, and **6**, respectively. Esterification of acid **2** with benzyl alcohol promoted by carbodiimide coupling reagents gave **5**, and acid-catalyzed esterification of **2** with *tert*-butanol using sulfuric acid gave **7**.

**Table 1:**
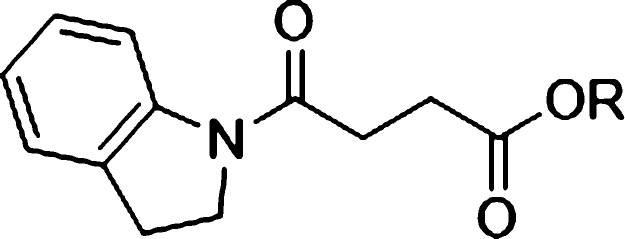
Notum Activities for 4-(Indolin-1-yl)-4-oxobutanoate Esters

compound	R	Notum OPTS^a^ IC_50_ (nM)	Notum TCF/LEF EC_50_ (nM)
**1**	Me	93 ± 20	530 ± 47
**2**	H (acid)	3100 ± 920	
**3**	Et	190 ± 45	300 ± 60
**4**	CH_2_CHF_2_	12 ± 2.8	
**5**	CH_2_Ph	64 ± 14	
**6**	CH(CH_3_)_2_	4300 ± 400	
**7**	C(CH_3_)_3_	inactive^b^	

a All values are mean ± s.d. (*n* = 4), experiments quoted to 2 s.f.

b <10% I @ 10 μM.

The Notum inhibitory potencies of these compounds were assessed by determining OPTS IC_50_ values, which can be used to compare the relative activities of covalent inhibitors under carefully regulated conditions.^43^ While **1** exhibits an IC_50_ of about 100 nM, the ethyl ester head replacement **3** has an IC_50_ of approximately double. However, when two further fluoride atoms are added, the 2,2-difluoroethyl ester **4** exhibits the best IC_50_ value (about 10 nM), indicating that the two fluoride atoms may help in positioning the molecule ideally for the first step of warhead hydrolysis. When a phenyl ring is added to produce benzyl ester, **5**, the IC_50_ is also slightly better than **1**. Adding methyl groups to the α-carbon of the ester worsens the IC_50_ substantially for **6**, while **7** is inactive. While this represents only a limited set of compounds, the results suggest that the activity is favored by electron-withdrawing groups and the lack of steric bulk in vicinity of the ester α-carbon.

Selected compounds were further investigated for inhibition of Notum activity in a Wnt/β-catenin signaling pathway TCF/LEF reporter (luciferase) HEK293 cell line with exogenous Wnt3a and Notum.^22,25^ Compound **1** gave an EC_50_ value of 530 nM, while **4** gave an EC_50_ of 300 nM, consistent with the more potent inhibition observed in the OPTS assay (Table 1).

We then performed Notum crystal soaking and subsequent crystallographic data collection for each compound (**3**–**7** in Table 1) that yielded complex structures with **3**, **4**, and **6** (Figure 4D–F). The complex structures were all determined at high resolution (1.2–1.4 Å, Supporting Information, Table S1) and show well-ordered electron densities for **3** and **4** (Figure 4D–E), with relatively weak density for **6** at a high contour level of 5σ (Figure 4F), which may be due to some warhead steric clashing. This is in agreement with its poor IC_50_ value. Curiously, no electron density was observed for **5**, despite its low IC_50_ value, this is possibly due to solubility issues. The *t*-butyl ester **7** also failed to show evidence of binding in the crystal soaking experiment, consistent with its absence of Notum inhibitory activity. This is presumably due to steric hindrance caused by the large *t*-butyl group. Superposition of the **1**-, **3**-, **4**-, and **6**-bound Notum structures shows that all four compounds possess an identical binding mode regardless of the warhead variations (Figure 4G). As expected, all the functional warheads were hydrolyzed at the predicted ester bond (Figure 4D–F). These data suggest that minimal steric bulk immediately adjacent to the ester C(O)O– bond is important for activity with all groups containing a −CH_2_– showing good activity. Electron-withdrawing groups beta to the ester enhanced activity, probably through activation of the ester to nucleophilic attack by S232. Increasing the steric bulk at the α-carbon of the ester by sequentially introducing additional methyl groups significantly reduced activity to the point where all activity was lost with the *t*-butyl ester (**3**: CH_2_CH_3_ > **6**: CH(CH_3_)_2_ ≫ **7**: C(CH_3_)_3_).

### Notum Covalent Inhibition Mechanism

**Figure 5:**
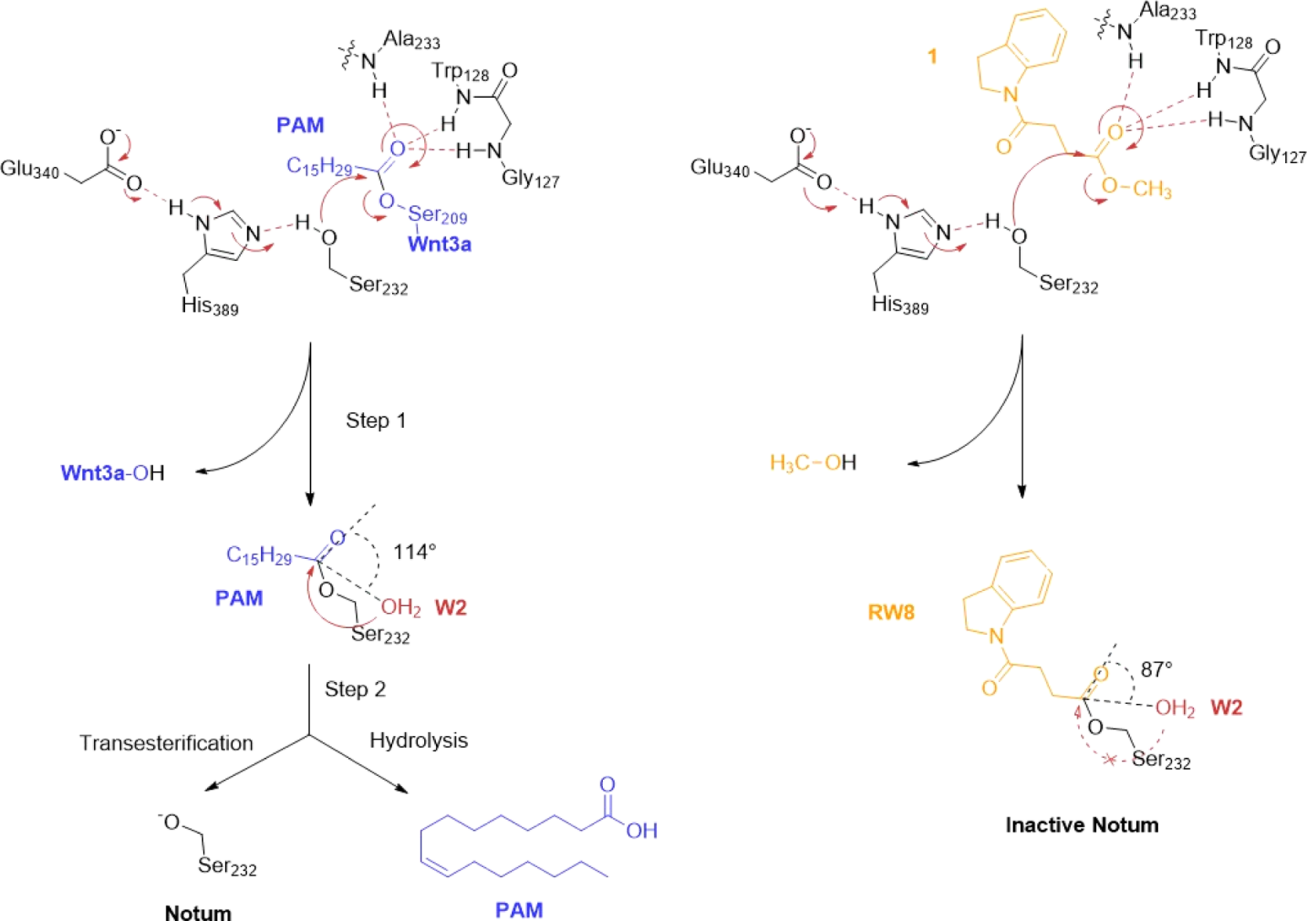
(A) Schematic presentation of Notum catalytic intermediates and final hydrolysis. (B) Inhibitor **1** acyl-enzyme forming step and covalent inhibition mechanism.

Notum, like many other carboxylesterases,^44^ catalyzes reactions by forming a covalent acyl-enzyme intermediate. This kind of intermediate is unstable and will be hydrolyzed and released, allowing the next substrate to dock. The Notum substrate catalysis mechanism proposed by us is schematically illustrated in Figure 5A. Typically, structural capture of a native substrate acyl-enzyme intermediate is difficult. However, if a substrate mimic has stronger hydrophobic interactions with the enzyme pocket residues, or it is positioned unfavorably toward a catalytic component, the hydrolysis/release of acyl-enzyme intermediate becomes inefficient (Figure 5B) and makes the structural capture of such an intermediate possible. This type of acyl-enzyme intermediate structure can help to elucidate the enzyme catalysis or inhibition mechanisms. We used the structure of Notum in complex with 1 as an example to explore the mechanism at the atomic level.

**Figure 6:**
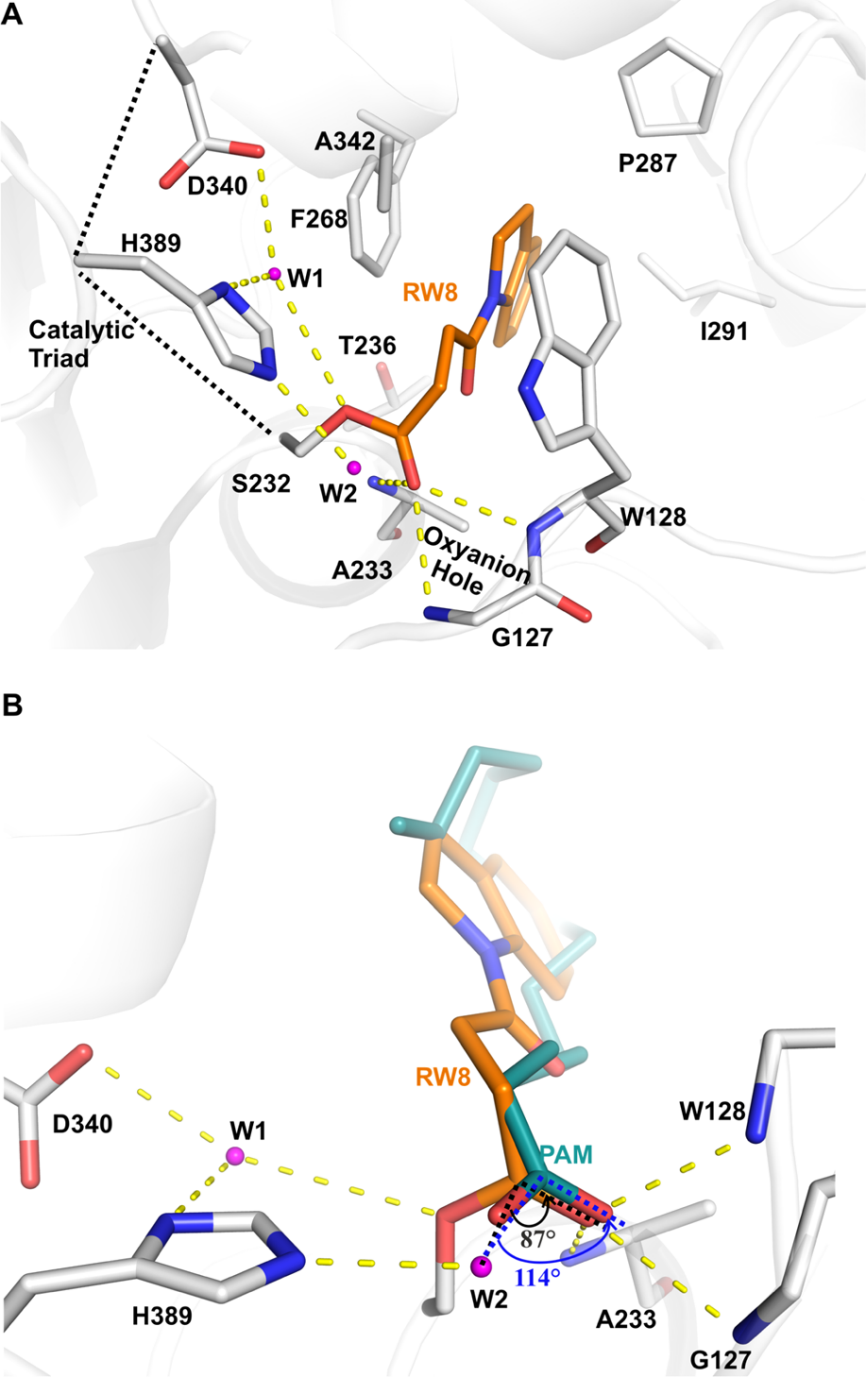
(A) Close-up view of **1** inhibitory acyl-enzyme intermediate (RW8, orange) interaction with Notum residues (gray sticks). Two active site waters (W1 and W2) are shown as magenta balls. Hydrogen bonds are shown as yellow dashed lines. (B) Close-up view of active site water 2 (W2) with its angle toward the carbonyl atom of the intermediate (PDB code 7ARG) and aligned with the substrate PAM structure complex (in teal, PDB code 4UZQ).

The complex structure shows hydrophobic interactions of **1** with T236, P287, I291, and A342 and more importantly the indole ring-forming hydrophobic stacking interactions with F268 and W128 (Figure 6A). These hydrophobic interactions are presumably stronger than those with the natural substrate PAM, which may hinder the release of **1**, whereby the acyl-enzyme intermediate is further hydrolyzed to the acid form. Our complex structures of the acid form **2** with Notum or its mutant demonstrate that the indole ring forms strong hydrophobic interactions with the Notum pocket residues and remains within the pocket (Figure 4A,B). In the **1** complex structure, there are two active site waters (Figure 6). Water 1 (W1) may coordinate protonation/deprotonation of the catalytic triad (D340, H389, and S232) which triggers nucleophile S232 attacking the carbonyl carbon of **1**, the formation of the acyl-enzyme intermediate, and release of the hydroxyl group.

The **1** head carbonyl oxygen interacts with A233, G127, and the W128 main chain amide NH groups, acting as hydrogen-bond donors to the scissile bond oxyanion (Figure 6A).

The second water (W2, Figure 6) is a nucleophile water recruited by H389 (forms hydrogen bond), with the ideal distance to the acyl-enzyme adduct carbonyl atom (2.9 Å). For efficient cleavage of the scissile ester bonds, H389 deprotonates this active site water, which then attacks the carbonyl carbon of the acyl enzyme. This active site water needs to be positioned at a suitable angle, the so-called BÜRGI angle,^45^ which is ideally around 107°. However, in the structure of Notum with **1**, the active site W2 is positioned at an angle of 87° toward the carbonyl carbon of the intermediate, which may be too small. This may explain why the **1** acyl-enzyme intermediate scissile ester bond cannot be further processed efficiently. Together with the strong hydrophobic interactions with the pocket, **1** becomes a covalent Notum inhibitor rather than a releasable substrate. When superposed with the natural substrate-bound Notum (S232A mutant form, PDB code 4UZQ), W2 is angled at 114° toward the natural substrate carbonyl atom (Figure 6B), close to the ideal angle. This suggests that W2 may be able to efficiently hydrolyze the natural PAM moiety acyl-enzyme intermediate. This carbonyl atom positional difference may be important for **1** as a covalent inhibitor.

## Conclusions

We identified 4-indolinyl-4-oxobutanoate and its ester derivatives as covalent Notum inhibitors. High-resolution complex structures with the designed compounds reveal a uniformly hydrolyzed ester form which covalently binds to the nucleophile serine and results in an identical structure regardless of different functional warheads. Mass spectrometry analysis of the Notum inhibitor complex under more physiologically relevant conditions displayed the expected mass increase, in agreement with our crystal structure observations. Mechanistically, an active site water positioned unfavorably for the inhibitor’s acyl-enzyme intermediate, together with its stronger hydrophobic interactions, may contribute to Notum covalent inhibition. This structural information may provide a guide for developing better Notum inhibitors.

## Experimental Section

### Docking, Compound Library, and Chemical Synthesis

A virtual library of approximately 1.5 million compounds from ChemDiv (San Diego CA, US) was filtered to match: MW (200–500); TPSA (20–120); HBD (≤ 2); log *D* (−4–5); NRB (<10); and ring assembly atom size (<13). Compounds with an MPO^46^ score <3.5, an SFI^47^ > 7, or containing potentially reactive groups^48^ were also removed. The remaining 534,804 compounds were subjected to docking with Schrödinger Glide SP (version 80012) using the previously published Notum structure (PDB code 6T2K, with waters deleted). The grid for docking was generated using the Glide Receptor Grid Generation tool. Those of docking score −9 or better (1330 compounds) were subject to manual inspection and availability check. A total of 952 compounds were purchased and experimentally screened using a cell-free OPTS biochemical assay.^22^ A total of 31 compounds with Notum IC_50_ < 500 nM were subject to Notum crystal soaking. Chemical synthesis details and purity HPLC traces are included in Supporting Information. Purity of compounds **1**–**7** was evaluated by NMR spectroscopy and LCMS analysis; all compounds had purity ≥95%.

### Notum Protein Expression, Purification, and Crystallization

A human Notum (UniProtKB ID: Q6P988) enzyme core sequence comprising amino acids S81–T451 with a C330S mutation was cloned into a stable cell line vector pNeo_sec.^49^ A stable HEK293S GNTI- cell line^50^ was used for protein production for crystallization. For functional assays, glycosylated protein was expressed in HEK293T cells. HEK cells were expanded and grown in roller bottles (Greiner). The conditioned medium was dialyzed and passed through a 5 ml HisTrap Excel column (GE Healthcare), followed by 20 mM imidazole PBS wash. Notum protein was eluted with 300 mM imidazole PBS and further purified by size-exclusion chromatography (Superdex 200 16/60 column, GE Healthcare) in 10 mM Hepes, pH 7.4, and 150 mM NaCl buffer. To remove flexible glycans to aid crystallization, the protein expressed in HEK293S GNTI- cells was deglycosylated with Endo F1 (*endo*-β-*N*-acetylglucosaminidase F1) at 37 °C, 1 h.^51^ For crystallization, deglycosylated Notum was concentrated to 5 mg/mL and crystallized in 96-well Swissci/MRC plates using the sitting drop vapor diffusion method^52^ at 21 °C. The crystallization drops contained 200 nL of Notum protein and 100 nL of reservoir solution of 1.5 M ammonium sulphate and 0.1 M sodium citrate, pH 4.2.

### Thermal Shift Assay

Thermal shift assays were carried out in a semiskirted 96-well PCR plate (4-Titude). Each well contains 3 μg of glycosylated Notum protein, 3× SYPRO Orange dye (Thermo Fisher Scientific), and compounds at various concentrations and adjusted to a final volume of 50 μL with assay buffer (10 mM Hepes, pH7.4, 150 mM NaCl, and 2% DMSO). The samples were heated in an Mx3005p qPCR machine (Stratagene, Agilent Technologies) from room temperature at a rate of 1 °C/min for 74 cycles. Fluorescence changes were monitored with excitation and emission wavelengths at 492 and 610 nm, respectively.

### Electrospray Mass Spectrometry

Reversed-phase chromatography was performed in-line prior to mass spectrometry using an Agilent 1290 uHPLC system (Agilent Technologies inc. USA). Concentrated protein samples were diluted to 0.02 mg/mL in 0.1% formic acid and 50 μL was injected on to a 2.1 mm × 12.5 mm Zorbax 5 μm 300SB-C3 guard column housed in a column oven set at 40 °C. The solvent system used consisted of A: 0.1% formic acid in ultrahigh-purity water (Millipore) and B: 0.1% formic acid in methanol (LC–MS grade, Chromasolve). Chromatography was started in 90% A and 10% B at a flow rate of 1.0 mL/min. A linear gradient from 10% B to 80% B was applied over 35 s. Elution then proceeded isocratically at 95% B for 40 s followed by equilibration under initial conditions for further 15 s. Protein intact mass was determined using a 6530 ESI-QTOF mass spectrometer (Agilent Technologies Inc. USA). The ion source was operated with the capillary voltage at 4000 V, nebulizer pressure at 60 psig, drying gas at 350 °C, and drying gas flow rate at 12 L/min. The instrument ion optic voltages were as follows: fragmentor 250 V, skimmer 60 V, and octopole RF 250 V.

### Notum OPTS Activity Assay

The OPTS activity assay has been described in previous reports.^24,25^ Briefly, the test compounds, the reporter substrate OPTS (Sigma), and the recombinant Notum protein were dispensed into 384-well plates (Greiner) using a Labcyte Echo 550 acoustic liquid handler and incubated 40 min in room temperature. The endpoint fluorescence was measured on a PheraSTAR FSX microplate reader with an excitation wavelength of 485 nm and an emission wavelength of 520 nm. The compound IC_50_ values were calculated from curves using a 4PL fit.

### Cell-based TCF/LEF Reporter (Luciferase) Assay

The cellular Wnt signaling functional assay has been described in previous reports.^24,25^ Briefly, the reporter cell plate containing stable HEK293 STF cells^53^ (1 × 10^4^ cells per well in 384-well microplates) carrying the Super Top Flash firefly luciferase reporter was prepared (overnight at 37 °C). Compounds and Notum protein were mixed for 10 min before the recombinant Wnt-3A was added and incubated for 1 h at room temperature. Then, the mixture from the compound plates was added to reporter cell plates and incubation overnight at 37 °C. For luciferase assay, steady-glo luciferase assay buffer (20 μL, Promega) was applied to the cell plates using the CyBio, the luminescence was measured on a PHERAstar FSXmicroplate reader with an excitation wavelength of 458 nm and an emission wavelength of 520 nm.

### Crystal Soaking, Data Collection, and Structural Analysis

For crystal soaking, compounds were dissolved in dimethyl sulfoxide (100 mg/mL) and then diluted into reservoir solution with 40% ethylene glycol at a concentration about 5 mg/mL. Equal amounts of compound solutions at concentrations of around 20 mM were applied to crystal drops for 30 min at room temperature. Crystals were flash-frozen in liquid nitrogen. Data sets were recorded from crystals at 100 K at the Diamond Light Source (I03), processed using Xia2,^54^ and refined with Refmac.^55^ The pymol Molecular Graphics System (Schrödinger, LLC, Cambridge, Cambridgeshire, UK) was used to prepare the figures.

## Supporting Information Available

Data collection and refinement statistics, chemical synthesis, and UPLC traces for lead compounds (PDF)
Molecular formula strings (CSV)

## Accession Codes

Coordinates for X-ray structures of Notum crystallized with **1** (PDB code 7ARG), **2** (PDB code 7B37), **3** (PDB code 7B2V), **4** (PDB code 7B2Y), **6** (PDB code 7B2Z), and Notum S232A mutant with **2** (PDB code 7B3F) have been deposited in the Protein Data Bank. Authors will release the atomic coordinates upon article publication.

## Abbreviations

ASU: asymmetric unit
Endo F: *endo*-β-*N*-acetylglucosaminidase F1
ESI-QTOF: electrospray ionization quadrupole time-of-flight
HPLC: high-performance liquid chromatography
OPTS: trisodium 8-octanoyloxypyrene-1,3,6-trisulfonate
PAM: palmitoleic acid
rmsd: root-mean-square deviation
TCF/LEF: T-cell factor/lymphoid enhancer-binding factor 1
uHPLC: ultrahigh-performance liquid chromatography
