# Supplementary material for: Structural Insights into Notum Covalent Inhibition
Source: J Med Chem. 2021 Jul 22;64(15):11354–63. doi: 10.1021/acs.jmedchem.1c00701 (PMC8365597; doi:10.1021/acs.jmedchem.1c00701)
Supplement: Supplementary file 1 — jm1c00701_si_001.pdf [file jm1c00701_si_001.pdf]

SUPPORTING INFORMATION

Structural Insights into Notum Covalent Inhibition

Yuguang Zhao,[1,‖] Fredrik Svensson,[2,‖] David Steadman,[2,‖] Sarah Frew,[2] Amy Monaghan,[2] Magda Bictash,[2] Tiago Moreira,[3] Rod Chalk,[3] Weixian Lu,[1] Paul V. Fish,[2,*] E. Yvonne Jones.[1,*]

[1]Division of Structural Biology, Wellcome Centre for Human Genetics, University of Oxford, Oxford, OX3 7BN, U.K.

[2]Alzheimer's Research UK UCL Drug Discovery Institute, University College London, Cruciform Building, Gower Street, London, WC1E 6BT, U.K.

[3]Centre for Medicines Discovery, University of Oxford, Oxford, OX3 7DQ, U.K.

*Corresponding authors: E-mail: PVF: p.fish@ucl.ac.uk, EYJ: yvonne@strubi.ox.ac.uk.

Table of Contents:

1 Data Collection and Refinement Statistics……………………………………2
2 Chemical Synthesis……………………………………………………...3
3 UPLC traces for lead compounds……………………………………………8

Table S1. Data Collection and Refinement Statistics

Notum complex with compounds	**1**	**2**	**2a***	**3**	**4**	**6**
PDB code	**7ARG**	**7B37**	**7B3F**	**7B2V**	**7B2Y**	**7B2Z**
Data collection	I03, Diamond Light Source (DLS)					
Wavelength (Å)	0.9762					
Space group	$P\ 2_1\ 2_1\ 2_1$					
Cell dimensions						
a, b, c (Å)	59.73, 71.78, 78.02	60.08, 71.53, 78.11	60.30, 71.26, 78.30	60.09, 71.53, 78.01	60.01, 71.54, 78.02	59.30, 73.02, 79.46
α, β, γ (°)	90, 90, 90					
Resolution (Å)	59.73 - 1.24 (1.26-1.24)†	78.12-1.34 (1.36-1.34)	47.8 - 1.39 (1.41-1.39)	52.7 - 1.24 (1.26-1.24)	52.7 - 1.23 (1.25 - 1.23)	52.9 - 1.24 (1.26 - 1.24)
I /σ(I)	16.8 (1.7)	16.7 (1.2)	11.7 (1.0)	16.4 (1.0)	19.9 (1.0)	19.6 (1.0)
CC 1/2	1.00 (0.75)	1.00 (0.38)	1.0 (0.46)	1.0 (0.5)	1.0 (0.6)	1.0 (0.8)
Unique reflections	95543 (9431)	98236 (4825)	68561 (3349)	95831 (4726)	97995 (4782)	95918 (4739)
Completeness (%)	99.7 (99.2)	100 (100)	100 (98.7)	100.0 (99.7)	100 (99.6)	100 (99.8)
Redundancy	39.5 (38.2)	39.5 (36.2)	13.0 (12.0)	39.6 (35.7)	39.6 (37.0)	25.9 (25.6)
**Refinement**						
R_{work} / R_{free}	0.181/0.198	0.195/0.202	0.189/0.219	0.184/0.194	0.186/0.207	0.213/0.226
Bond length (Å)	0.009	0.007	0.010	0.016	0.016	0.008
Bond angle (°)	1.15	1.00	1.18	1.51	1.52	1.18
Average B ($Å^2$)	28	20	24	22	22	26

a* indicate the S232A mutant Notum used. †Values in parentheses are for highest-resolution shell.

Chemical Synthesis

All reagents and solvents were purchased from commercial suppliers and used without further purification. Thin layer chromatography (TLC) was carried out on aluminium backed silica plates. The plates were visualized under UV (254 nm) light, followed by staining with phosphomolybdic acid dip or potassium permanganate and gentle heating. Organic solvent layers were routinely dried with anhydrous $MgSO_4$ or with a Biotage ISOLUTE phase separator and concentrated using a Büchi rotary evaporator. Compound purification by column chromatography was performed using a Biotage Isolera using prepacked Biotage SNAP KP-Sil silica cartridges. ^1H and ^{13}C NMR spectra were run in deuterated (≥99.5%) solvents on either a Bruker Avance 600 (600 MHz) or Bruker Avance 700 (700 MHz). Chemical shifts (δ) are reported as parts per million (ppm), coupling constants (*J*) are reported in Hz and signal multiplicities are reported as singlet (s), doublet (d), triplet (t), quartet (q), doublet of doublets (dd), triplet of doublets (td), triplet of triplets (tt), multiplet (m), or broad singlet (br s). LCMS analysis was performed on a Waters Acquity H-Class UPLC system using an acidic (HSS C18 Column, H_2O:MeCN, 0.1% CF_3CO_2H) mobile phase. Purity of compounds **1**-**7** was evaluated by NMR spectroscopy and LCMS analysis; all compounds had purity ≥ 95 %.

The original hit methyl 4-(indolin-1-yl)-4-oxobutanoate (**1**) was purchased from ChemDiv (San Diego CA, US; https://www.chemdiv.com/) (cat. no. 2663-0001) as a stock solution (10 mM in DMSO) and then resynthesized as a solid sample to confirm structure and purity.

Methyl 4-(indolin-1-yl)-4-oxobutanoate (**1**):

A solution of methyl 4-chloro-4-oxobutanoate (37.9 mg, 0.25 mmol), indoline (0.02 mL, 0.21 mmol) and 4-(dimethylamino)pyridine (5.1 mg, 0.04 mmol) in pyridine (1 mL) was stirred at 50 °C for 3 h. The reaction was then quenched with water, extracted with CH_2Cl_2 and the combined organic layers were dried and evapourated. Purification by chromatography (0-100% EtOAc:cyclohexane) gave **1** as a white solid (30 mg, 61 %).

^{1}H NMR (600 MHz, $CDCl_3$) δ 8.19 (d, J = 8.3 Hz, 1H), 7.26 – 7.13 (m, 2H), 7.00 (t, J = 7.4 Hz, 1H), 4.09 (t, J = 8.5 Hz, 2H), 3.71 (s, 3H), 3.21 (t, J = 8.5 Hz, 2H), 2.75 (m, 4H). ^{13}C NMR (151 MHz, $CDCl_3$) δ 173.66, 169.53, 143.01, 131.13, 127.68, 124.67, 123.81, 117.09, 52.06, 47.95, 30.78, 28.76, 28.16. LCMS: t_R 1.61 min, m/z 234.2 $[M + H]^+$.

4-(Indolin-1-yl)-4-oxobutanoic acid (**2**):

To a stirred solution of methyl 4-(indolin-1-yl)-4-oxo-butanoate (**1**) (30.0 mg, 0.13 mmol) in MeOH (2 mL), THF (1 mL), H_2O (1 mL) was added aqueous LiOH (5mL, 1 M) and the reaction was stirred overnight at RT. The reaction was then acidified with dilute HCl (2 M) and extracted with CH_2Cl_2. The combined organic layers were passed through a phase separator and concentrated to give **2** as a pale orange solid (19 mg, 67 %).

^{1}H NMR (600 MHz, $CDCl_3$) δ 8.19 (d, J = 8.2 Hz, 1H), 7.19 (t, J = 7.3 Hz, 2H), 7.03 (t, J = 7.3 Hz, 1H), 4.08 (t, J = 8.3 Hz, 2H), 3.22 (t, J = 8.3 Hz, 2H), 2.78 (m, 4H). ^{13}C NMR (151 MHz, $CDCl_3$) δ 176.99, 169.93, 142.73, 131.23, 127.76, 124.75, 124.16, 117.27, 48.04, 30.72, 29.17, 28.13. LCMS: t_R 1.50 min, m/z 220.1 $[M + H]^+$.

Ethyl 4-(indolin-1-yl)-4-oxobutanoate (**3**):

To a stirred solution of methyl 4-(indolin-1-yl)-4-oxo-butanoate (**1**) (100.0 mg, 0.43 mmol) in EtOH (2 mL) was added *p*-toluenesulfonic acid monohydrate (10.0 mg, 0.05 mmol) and the reaction was heated to 40 ºC overnight. The reaction was then concentrated to dryness and purified by chromatography (0-50% EtOAc:cyclohexane) to give **3** as an off-white solid (29 mg, 27 % yield).

^{1}H NMR (600 MHz, DMSO-d_6) δ 8.03 (d, J = 7.9 Hz, 1H), 7.23 (d, J = 7.4 Hz, 1H), 7.13 (t, J = 7.9 Hz, 1H), 6.98 (t, J = 7.4 Hz, 1H), 4.14 – 4.03 (m, 4H), 3.15 (t, J = 8.5 Hz, 2H), 2.71 (t, J = 6.4 Hz, 2H), 2.58 (t, J = 6.4 Hz, 2H), 1.19 (t, J = 7.1 Hz, 3H). ^{13}C NMR (151 MHz, DMSO-d_6) δ 172.52, 169.65, 142.90, 131.67, 126.98, 124.86, 123.18, 115.80, 59.88, 47.21, 30.02, 28.41, 27.40, 14.14. LCMS: t_R 1.70 min, m/z 248.2 [M + H]$^+$.

2,2-Difluoroethyl 4-(indolin-1-yl)-4-oxobutanoate (**4**):

To a stirred solution of methyl 4-(indolin-1-yl)-4-oxo-butanoate (**1**) (72.0 mg, 0.31 mmol) in 2,2-difluoroethanol (0.5 mL, 7.9 mmol) was added *p*-toluenesulfonic acid monohydrate (4.0 mg, 0.02 mmol) and the reaction was heated to 40 ºC overnight. The reaction was then concentrated to dryness and purified by chromatography (0-50% EtOAc:cyclohexane) to give **4** as a colourless oil (20 mg, 23 % yield).

^{1}H NMR (700 MHz, DMSO-d_6) δ 8.02 (d, J = 7.9 Hz, 1H), 7.22 (d, J = 7.3 Hz, 1H), 7.12 (t, J = 7.9 Hz, 1H), 6.97 (t, J = 7.3 Hz, 1H), 6.25 (tt, J = 54.3, 3.3 Hz, 1H), 4.32 (td, J = 15.4, 3.3 Hz, 2H), 4.10 (t, J = 8.5 Hz, 2H), 3.14 (t, J = 8.5 Hz, 2H), 2.76 – 2.70 (m, 2H), 2.67 (m, 2H). ^{13}C NMR (176 MHz, DMSO-d_6) δ 171.94, 169.41, 142.86, 131.68, 126.97, 124.85, 123.23,

115.83, 113.53 (t, J = 238.9 Hz), 61.83 (t, J = 26.4 Hz), 47.22, 29.94, 28.12, 27.41. LCMS: t_R 1.76 min, m/z 284.1 $[M + H]^+$.

Benzyl 4-(indolin-1-yl)-4-oxobutanoate (**5**):

To a stirred solution of 4-(indolin-1-yl)-4-oxo-butanoic acid (**2**) (64.0 mg, 0.29 mmol) in DMF (1.5 mL) was added Et_3N (0.05 mL, 0.35 mmol), 4-(dimethylamino)pyridine (10.7 mg, 0.09 mmol) and *N*-(3-(dimethylamino)propyl)-*N'*-ethylcarbodiimide hydrochloride (WSCDI) (67.2 mg, 0.35 mmol). The mixture was stirred at RT for 10 min before benzyl alcohol (63.1 mg, 0.58 mmol) was added and the reaction heated to 70 °C overnight. The cooled reaction mixture was diluted with CH_2Cl_2 and then washed with dilute HCl (1 M) followed by sat. aqueous $NaHCO_3$. The organic layer was passed through a phase separator before being loaded onto Biotage ISOLUTE HM-N and purified by chromatography (0-20% EtOAc:cyclohexane) to give **5** as a clear oil which formed a white wax on standing (20 mg, 22 %).

^{1}H NMR (700 MHz, DMSO-d_6) δ 8.04 (d, J = 7.9 Hz, 1H), 7.38 – 7.29 (m, 5H), 7.22 (d, J = 7.3 Hz, 1H), 7.13 (t, J = 7.9 Hz, 1H), 6.97 (t, J = 7.3 Hz, 1H), 5.10 (s, 2H), 4.09 (t, J = 8.5 Hz, 2H), 3.13 (t, J = 8.5 Hz, 2H), 2.74 (t, J = 6.3 Hz, 2H), 2.66 (t, J = 6.3 Hz, 2H). ^{13}C NMR (176 MHz, DMSO-d_6) δ 172.39, 169.63, 142.91, 136.30, 131.68, 128.40, 127.94, 127.81, 126.98, 124.84, 123.20, 115.84, 65.44, 47.24, 30.05, 28.50, 27.41. LCMS: t_R 1.85 min, m/z 310.1 $[M + H]^+$.

Isopropyl 4-(indolin-1-yl)-4-oxobutanoate (**6**):

To a stirred solution of methyl 4-(indolin-1-yl)-4-oxo-butanoate (**1**) (100.0 mg, 0.43 mmol) in 2-propanol (2 mL) was added *p*-toluenesulfonic acid monohydrate (5.0 mg, 0.03 mmol) and

the reaction was heated to 40 °C overnight. The reaction was then concentrated to dryness and purified by chromatography (0-50% EtOAc:cyclohexane) to give **6** as a clear oil (56 mg, 50 %).

^{1}H NMR (600 MHz, DMSO-*d*$_6$) δ 8.03 (d, *J* = 8.0 Hz, 1H), 7.23 (d, *J* = 7.3 Hz, 1H), 7.13 (t, *J* = 8.0 Hz, 1H), 6.98 (t, *J* = 7.3 Hz, 1H), 4.88 (hept, 6.2 Hz, 1H), 4.10 (t, *J* = 8.5 Hz, 2H), 3.15 (t, *J* = 8.5 Hz, 2H), 2.70 (t, *J* = 6.4 Hz, 2H), 2.54 (t, *J* = 6.4 Hz, 2H), 1.18 (d, *J* = 6.2 Hz, 6H). ^{13}C NMR (151 MHz, DMSO-*d*$_6$) δ 171.99, 169.67, 142.92, 131.66, 126.98, 124.85, 123.16, 115.80, 67.10, 47.23, 30.03, 28.68, 27.40, 21.64. LCMS: t_R 1.82 min, *m*/*z* 262.2 $[M + H]^+$.

tert-Butyl 4-(indolin-1-yl)-4-oxobutanoate (**7**):

Concentrated sulfuric acid (0.03 mL, 0.57 mmol) was added to a vigorously stirred suspension of anhydrous $MgSO_4$ (272.3mg, 2.26 mmol) in CH_2Cl_2 (5 mL) in a thick-walled reaction vial. The mixture was stirred for 15 minutes and then 4-(indolin-1-yl)-4-oxo-butanoic acid (**2**) (124.0 mg, 0.57 mmol) was added followed by 2-methylpropan-2-ol (0.27 mL, 2.83 mmol). The vial was then sealed with a Teflon-lined crimp cap and stirred for 18 h at RT. The reaction mixture was basified with sat. aqueous $NaHCO_3$ and extracted with CH_2Cl_2. The combined organic layers were dried through a phase separator, concentrated and purified by chromatography (0-20% EtOAc:cyclohexane) to give **7** as a white solid (20 mg, 13 % yield).

^{1}H NMR (700 MHz, $CDCl_3$) δ 8.21 (d, *J* = 8.0 Hz, 1H), 7.18 (m, 2H), 7.00 (td, *J* = 7.4, 1.1 Hz, 1H), 4.10 (t, *J* = 8.5 Hz, 2H), 3.20 (t, *J* = 8.5 Hz, 2H), 2.68 (m, 4H), 1.46 (s, 9H). ^{13}C NMR (176 MHz, DMSO-*d*$_6$) δ 171.74, 169.74, 142.96, 131.65, 126.97, 124.83, 123.13, 115.82, 79.59, 47.28, 30.10, 29.54, 27.82, 27.42. LCMS: t_R 1.84 min, *m*/*z* 298.2 $[M + Na]^+$.

UPLC traces for lead compounds

1

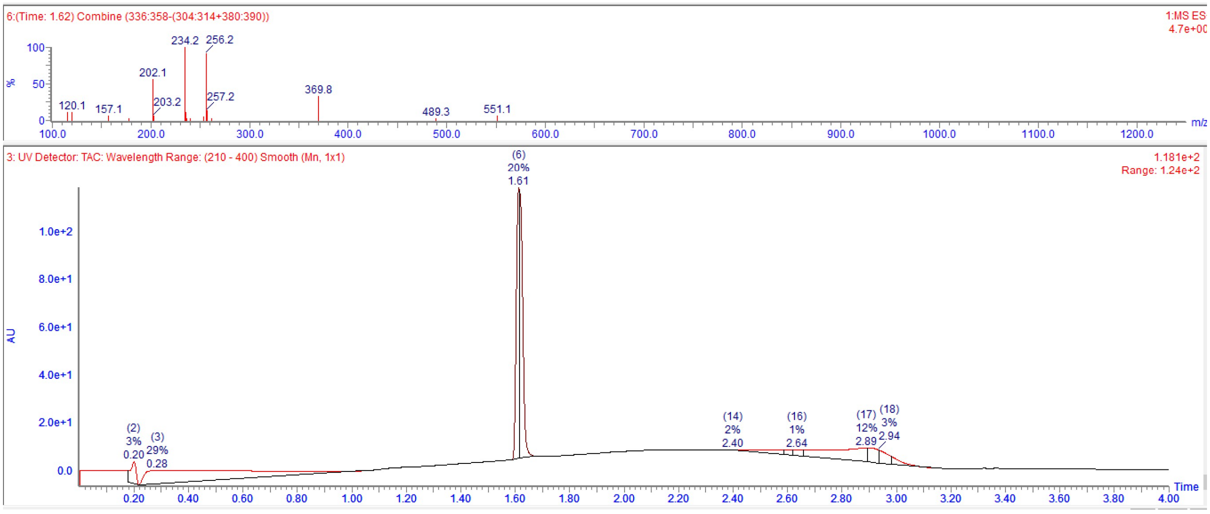

2

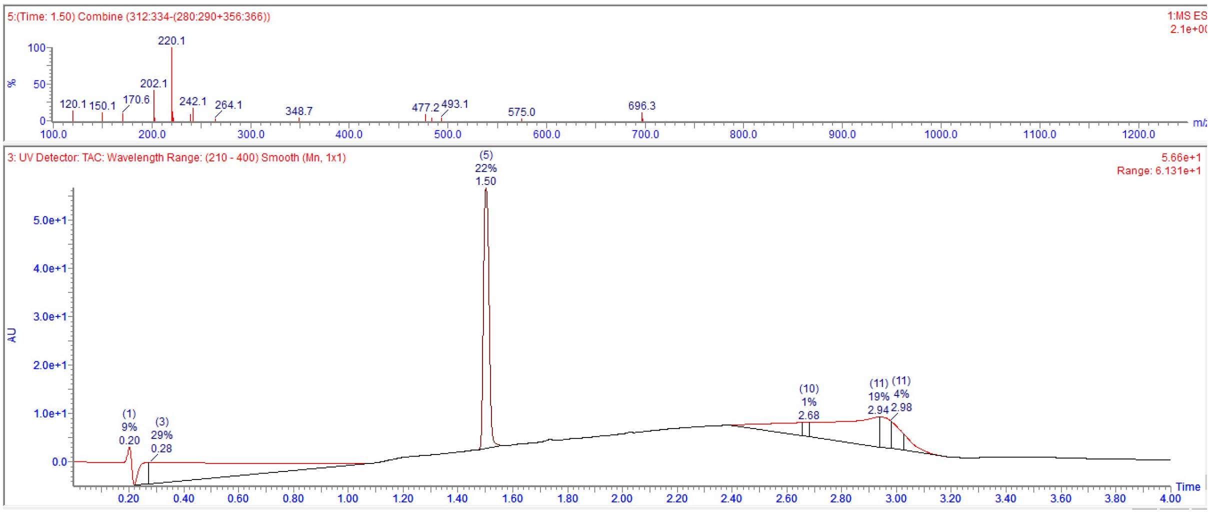

3

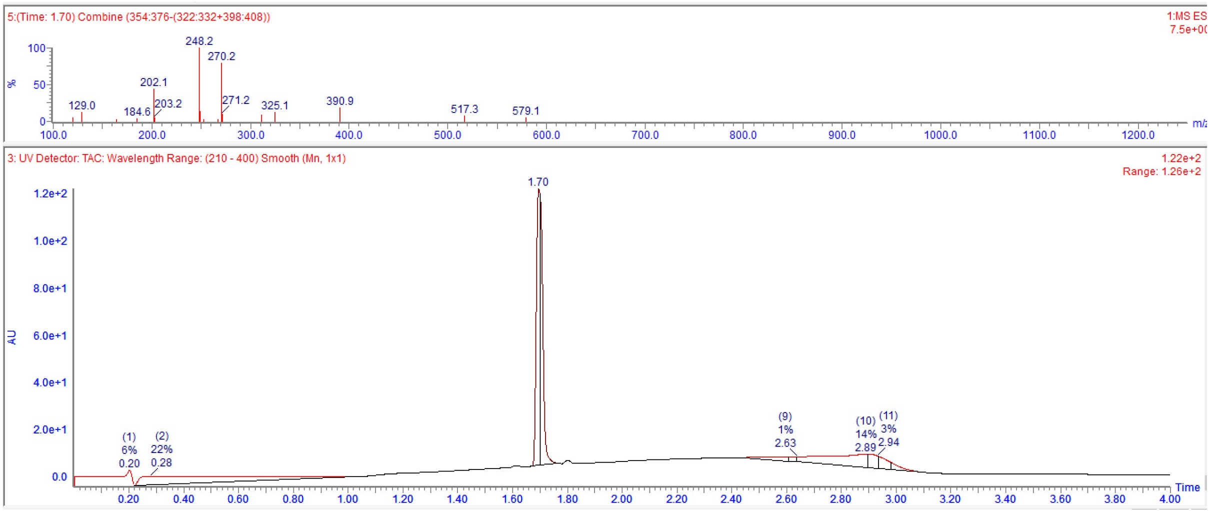

4

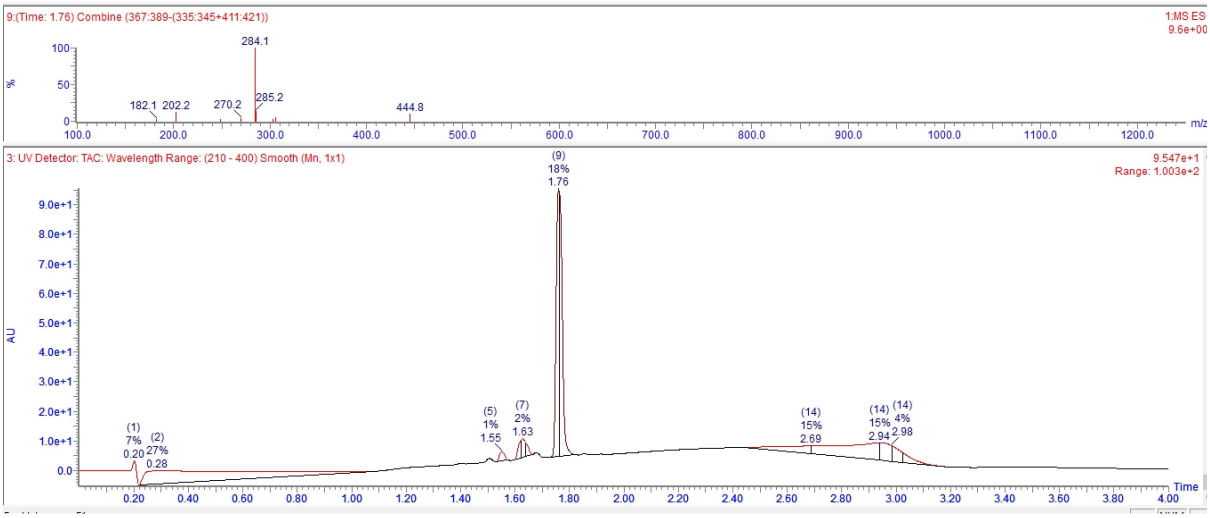
9:(Time: 1.76) Combine (367:389-(335:345+411:421))
1:MS ES-
9.6e+00
284.1
182.1
202.2
270.2
285.2
444.8
m/z
3: UV Detector: TAC: Wavelength Range: (210 - 400) Smooth (Mn, 1x1)
9.547e+1
Range: 1.003e+2
(9)
18%
1.76
AU
Time

5

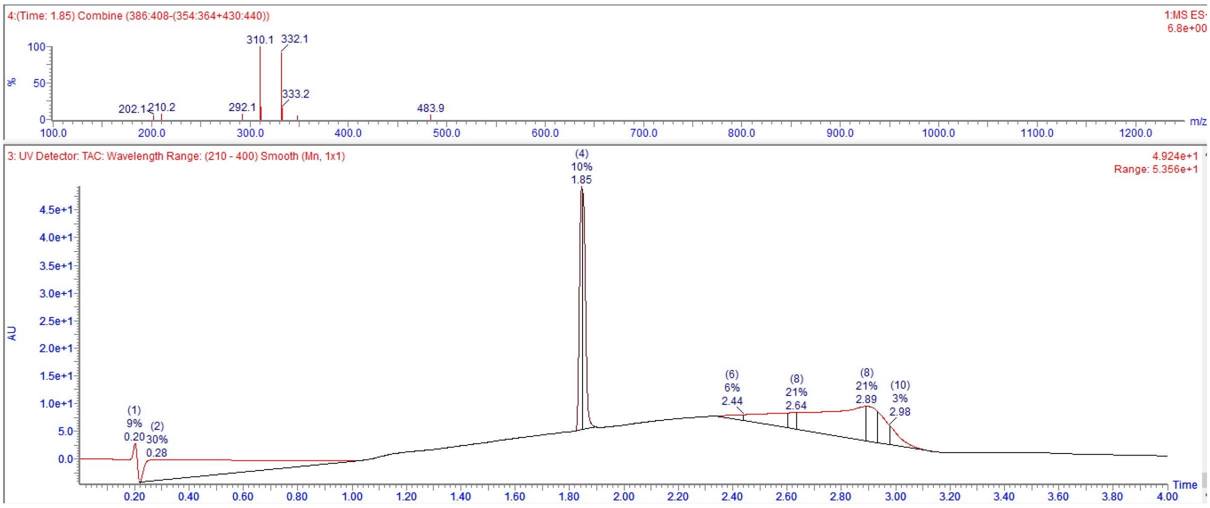
4:(Time: 1.85) Combine (386:408-(354:364+430:440))
1:MS ES-
6.8e+00
310.1
332.1
202.1
210.2
292.1
333.2
483.9
m/z
3: UV Detector: TAC: Wavelength Range: (210 - 400) Smooth (Mn, 1x1)
4.924e+1
Range: 5.356e+1
(4)
10%
1.85
AU
Time

6

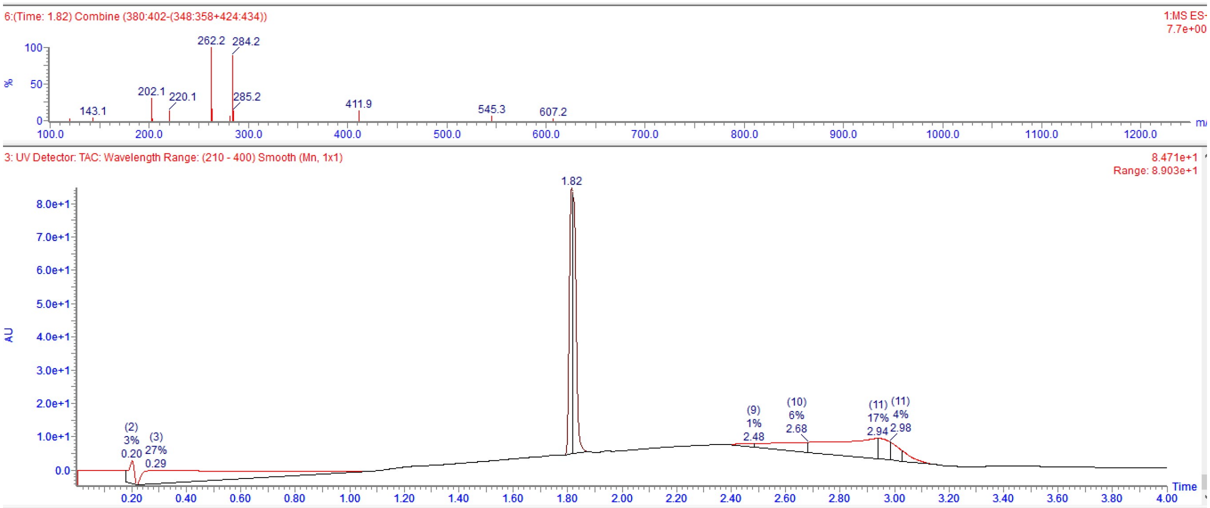
6:(Time: 1.82) Combine (380:402-(348:358+424:434))
1:MS ES-
7.7e+00
262.2
284.2
143.1
202.1
220.1
285.2
411.9
545.3
607.2
3: UV Detector: TAC: Wavelength Range: (210 - 400) Smooth (Mn, 1x1)
8.471e+1
Range: 8.903e+1
1.82
AU
Time

7

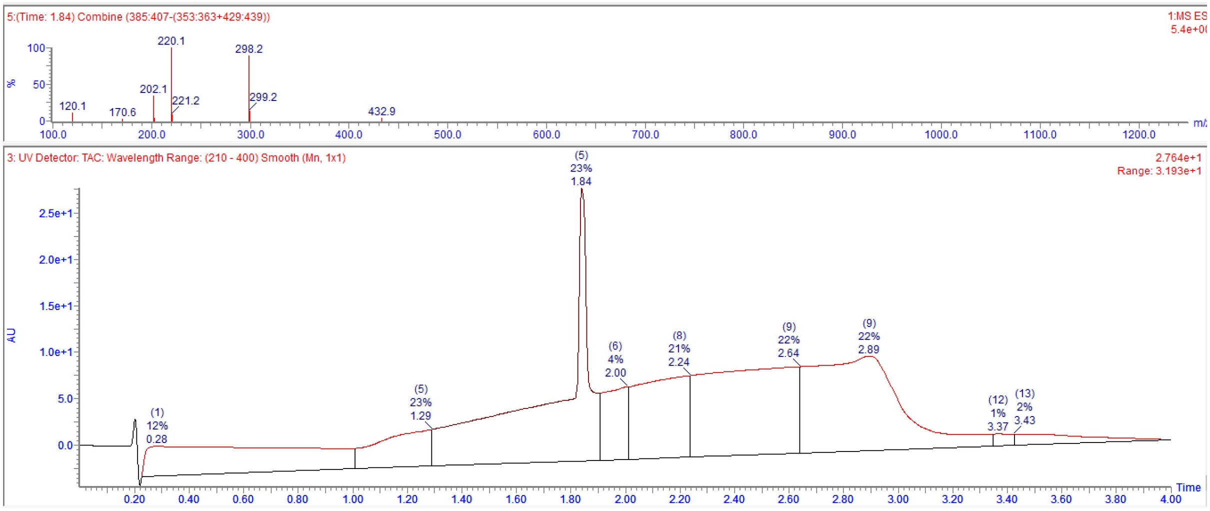
5:(Time: 1.84) Combine (385:407-(353:363+429:439))
1:MS ES
220.1
298.2
202.1
221.2
299.2
120.1
170.6
432.9
3: UV Detector: TAC: Wavelength Range: (210 - 400) Smooth (Mn, 1x1)
2.764e+1
Range: 3.193e+1
AU
Time
